# Recognition of Variants of Concern by Antibodies and T Cells Induced by a SARS-CoV-2 Inactivated Vaccine

**DOI:** 10.3389/fimmu.2021.747830

**Published:** 2021-11-09

**Authors:** Felipe Melo-González, Jorge A. Soto, Liliana A. González, Jorge Fernández, Luisa F. Duarte, Bárbara M. Schultz, Nicolás M. S. Gálvez, Gaspar A. Pacheco, Mariana Ríos, Yaneisi Vázquez, Daniela Rivera-Pérez, Daniela Moreno-Tapia, Carolina Iturriaga, Omar P. Vallejos, Roslye V. Berríos-Rojas, Guillermo Hoppe-Elsholz, Marcela Urzúa, Nicole Bruneau, Rodrigo A. Fasce, Judith Mora, Alba Grifoni, Alessandro Sette, Daniela Weiskopf, Gang Zeng, Weining Meng, José V. González-Aramundiz, Pablo A. González, Katia Abarca, Eugenio Ramírez, Alexis M. Kalergis, Susan M. Bueno

**Affiliations:** ^1^ Millennium Institute on Immunology and Immunotherapy, Pontificia Universidad Católica de Chile, Santiago, Chile; ^2^ Departamento de Genética Molecular y Microbiología, Facultad de Ciencias Biológicas, Pontificia Universidad Católica de Chile, Santiago, Chile; ^3^ Departamento de Laboratorio Biomédico, Instituto de Salud Pública de Chile, Santiago, Chile; ^4^ Departamento de Enfermedades Infecciosas e Inmunología Pediátrica, División de Pediatría, Escuela de Medicina, Pontificia Universidad Católica de Chile, Santiago, Chile; ^5^ Center for Infectious Disease and Vaccine Research, La Jolla Institute for Immunology (LJI), La Jolla, CA, United States; ^6^ Department of Medicine, Division of Infectious Diseases and Global Public Health, University of California, San Diego (UCSD), La Jolla, CA, United States; ^7^ Sinovac Biotech, Beijing, China; ^8^ Departamento de Farmacia, Facultad de Química y de Farmacia, Pontificia Universidad Católica de Chile, Santiago, Chile; ^9^ Departamento de Endocrinología, Facultad de Medicina, Escuela de Medicina, Pontificia Universidad Católica de Chile, Santiago, Chile

**Keywords:** CoronaVac, SARS-CoV-2, antibodies, vaccine, variants of concern, T cell immunity

## Abstract

**Background:**

Severe acute respiratory syndrome coronavirus 2 (SARS-CoV-2) is the virus responsible of the current pandemic ongoing all around the world. Since its discovery in 2019, several circulating variants have emerged and some of them are associated with increased infections and death rate. Despite the genetic differences among these variants, vaccines approved for human use have shown a good immunogenic and protective response against them. In Chile, over 70% of the vaccinated population is immunized with CoronaVac, an inactivated SARS-CoV-2 vaccine. The immune response elicited by this vaccine has been described against the first SARS-CoV-2 strain isolated from Wuhan, China and the D614G strain (lineage B). To date, four SARS-CoV-2 variants of concern described have circulated worldwide. Here, we describe the neutralizing capacities of antibodies secreted by volunteers in the Chilean population immunized with CoronaVac against variants of concern Alpha (B.1.1.7), Beta (B.1.351) Gamma (P.1) and Delta (B.617.2).

**Methods:**

Volunteers enrolled in a phase 3 clinical trial were vaccinated with two doses of CoronaVac in 0-14 or 0-28 immunization schedules. Sera samples were used to evaluate the capacity of antibodies induced by the vaccine to block the binding between Receptor Binding Domain (RBD) from variants of concern and the human ACE2 receptor by an in-house ELISA. Further, conventional microneutralization assays were used to test neutralization of SARS-CoV-2 infection. Moreover, interferon-γ-secreting T cells against Spike from variants of concern were evaluated in PBMCs from vaccinated subjects using ELISPOT.

**Results:**

CoronaVac promotes the secretion of antibodies able to block the RBD of all the SARS-CoV-2 variants studied. Seropositivity rates of neutralizing antibodies in the population evaluated were over 97% for the lineage B strain, over 80% for Alpha and Gamma variants, over 75% for Delta variant and over 60% for the Beta variant. Geometric means titers of blocking antibodies were reduced when tested against SARS-CoV-2 variants as compared to ancestral strain. We also observed that antibodies from vaccinated subjects were able to neutralize the infection of variants D614G, Alpha, Gamma and Delta in a conventional microneutralization assay. Importantly, after SARS-CoV-2 infection, we observed that the blocking capacity of antibodies from vaccinated volunteers increased up to ten times for all the variants tested. We compared the number of interferon-γ-secreting T cells specific for SARS-CoV-2 Spike WT and variants of concern from vaccinated subjects and we did not detect significant differences.

**Conclusion:**

Immunization with CoronaVac in either immunization schedule promotes the secretion of antibodies able to block SARS-CoV-2 variants of concern and partially neutralizes SARS-CoV-2 infection. In addition, it stimulates cellular responses against all variants of concern.

## Introduction

SARS-CoV-2 represents a global threat to public health and has been responsible for over 4 million deaths worldwide to date (1). After the spread of the original wild-type SARS-CoV-2 strain, multiple mutants have arisen around the world. Most of these circulating variants belong to the SARS-CoV-2 lineage B, in particular lineage B.1 (2). One of the most prevalent strains is the D614G, which displays a mutation in the C-terminal region of the Spike 1 (S1) domain outside the Receptor Binding Domain (RBD) (2). Although this mutant has been reported to be more infective, sera from convalescent patients and subjects vaccinated with mRNA vaccines are able to neutralize the D614G mutant to an extent similar to that of the ancestral strain, i.e. lineage B or wild type strain (2–5).

Current vaccination programs around the world are facing the threat of these circulating variants of concern of SARS-CoV-2, as they exhibit different mutations in the RBD and may evade antibody neutralization (2). To facilitate their identification, variants of concern are currently termed Alpha (B.1.1.7), Beta (B1.351), Gamma (P.1), and Delta (B.617.2) (6). Alpha (first identified in the UK), Beta (first identified in South Africa) and Gamma (first identified in Brazil) mutants share the N501Y mutation that has been linked with increased affinity of the Spike protein for the endogenous receptor human Angiotensin-converting enzyme 2 (hACE2) (7). Beta and Gamma mutants exhibit the E484K mutation, associated with an increased evasion of neutralizing antibodies (8–10). Furthermore, Beta and Gamma exhibit mutations in the residue K417 of the RBD but differ in the amino acid substitutions (K417N for Beta and K417T for Gamma), which may affect antibody binding (6). In addition, the Delta variant (first identified in India) is currently a cause of concern due to its high transmissibility and may even surpass other variants in this regard (11). Delta exhibits unique mutations (L452R, T478K and P681R), which may increase viral infectivity and viral fusion (12, 13). Considering the increased infectivity and death rates described for these variants, it is crucial to understand whether vaccination can induce protection against them (6).

Chile is among the countries with the highest percentage of vaccination worldwide (over 56% of the total population), and CoronaVac, an inactivated SARS-CoV-2 vaccine, represents 78.2% of the immunized population (14). A phase 3 clinical trial is being conducted in Chile, with two vaccination schedules: two doses separated by 14 days (0-14) or by 28 days (0-28), and the general population has received the latter schedule. CoronaVac is safe and induces humoral and cellular responses in vaccinated subjects from different age groups, and has been proven effective in remarkably reducing hospitalizations and death rates (15, 16). Here, we evaluate the blocking and neutralizing capacities of circulating antibody induced by CoronaVac in vaccinated volunteers for both schedules against the most prevalent variants in Chile. Blocking capacities against the RBD of variants Alpha, Beta, Gamma and Delta were tested with an in-house surrogate neutralization test (sVNT) and compared to the wild strain, included in the vaccine formulation. The neutralizing capacities of antibody were evaluated using a conventional plaque-reduction neutralization test (cVNT) for the D614G, Alpha, Gamma and Delta variants. Our data shows that vaccinated volunteers exhibit circulating antibodies with neutralizing capacities against the different variants of concern, with a better response against the Alpha and Gamma variants, although inhibition of the binding between hACE2 and RBD from the Beta variant was also detected using sVNT. We also observed that CoronaVac promotes Interferon-y (IFN-γ)-producing CD4^+^ T cells against Spike peptides from variants of concern. These results suggest that the antibodies and cellular responses induced by the administration of two doses of CoronaVac would have a protective role against the several circulating variants of concern of SARS-CoV-2.

## Methods

### Study Design and Volunteers

The clinical trial (clinicaltrials.gov NCT04651790) was conducted in Chile at eight different sites and evaluated two immunization schedules. This trial was approved by each Institutional Ethical Committee and the Chilean Public Health Institute (#24204/20) and conducted according to the current Tripartite Guidelines for Good Clinical Practices, the Declaration of Helsinki (17), and local regulations. Volunteers were inoculated with either two doses of 3 µg (600SU) of CoronaVac at 0- and 14-days or 0- and 28-days post the first immunization (p.i.). Written informed consent was obtained from each participant. Exclusion criteria included history of confirmed symptomatic SARS-CoV-2 infection, pregnancy, allergy to vaccine components, and immunocompromised conditions. A complete list of inclusion and exclusion criteria has been published previously (15). A total of 2,302 volunteers were enrolled by March 19^th^, 2021, and a subgroup of 440 volunteers was chosen to evaluate their immune response. Demographic information, co-morbidities, nutritional status, immunization schedule, and dates of vaccination, were obtained at enrolment for all volunteers.

### Procedures

Sera samples from the 0-14 and 0-28 immunization schedules were chosen among those that were previously confirmed as positive against wild-type SARS-CoV-2 through commercial kits (GenScript #L00847-A and BioHermes #COV-S41). A total of 42 samples (22 samples from the 0-14 schedule and 20 from the 0-28 schedule) were evaluated by sVNT. A total of 52 samples (34 samples from the 0-14 schedule and 18 samples from the 0-28 schedule) were evaluated by cVNT. Both groups included volunteers aged 18 to 59 years and over 60 years.

To assess the capacity of the antibodies against SARS-CoV-2 circulating variants of concern to inhibit RBD and hACE2 interaction in the samples from vaccinated volunteers, we performed in-house SARS-CoV-2 sVNT based on previous reports (18). RBD unconjugated proteins from wild-type (WT) SARS-CoV-2 (GenScript #Z03483) and the variants B.1.1.7 (GenScript #Z03533), B.1.351 (GenScript #Z03537) P.1 (SinoBiological #40592-V08H86) and B.1.617.2 (GenScript #Z03613) were conjugated to HRP using the HRP Conjugation Kit - Lightning Link (#ab102890) in a 2:1 mass ratio (HRP to RBD) following the instructions of the manufacturer. ELISA 96-well plates (SPL) were pre-coated with 100 ng per well of the recombinant hACE2 protein (GenScript #Z03484) in 50 μL of 100 mM carbonate–bicarbonate coating buffer (pH 9.6) ON at 4°C. Plates were then washed three times with PBS - 0.05% Tween 20 and blocked with PBS - 10% FBS for 2h at RT. The HRP-RBD conjugates obtained previously were then incubated with the serum sample in a final volume of 120 µL for 1 h at 37°C. Concentration of conjugates used were as follows: 3 ng of WT SARS-CoV-2, 0.75 ng of B.1.1.7, 3 ng of B.1.351, 3 ng of P.1 and 3 ng of B.1.617.2. Then, these mixtures were added into the 96-well plates coated with hACE2 and were incubated for 1 h at RT. Unbound HRP-RBD were removed washing five times with PBS - 0.05% Tween 20. Then, 50 µL of 3,3’,5,5’-tetramethylbenzidine (TMB – BD #555214) was added. An equal volume of 2 N H_2_SO_4_ was added to stop the reaction, and optical densities (OD) values at 450 nm were read. The antibody titer was determined as the last fold-dilution with a cut-off value over 20% of inhibition. The percentage of inhibition was defined as: [OD_450nm_ value of negative control-OD_450nm_ value of sample]/[OD_450nm_ value of negative control*100]. Negative controls (corresponding to sera sample obtained before immunization) were included. For the cVNT, sera samples were two-fold serially diluted starting at a 4-fold dilution until a 512-fold. Then, samples were incubated for 1 h at 37°C with an equal volume of a SARS-CoV-2 33782CL-SARS-CoV-2 strain (lineage B, D614G), Alpha (B.1.1.7), Gamma (P.1) and Delta (B.1.617.2) variants. These variants were previously isolated by the Institute of Public Health of Chile from clinical samples. These mixtures were inoculated on confluent Vero E6 cell monolayers (ATCC CRL-1586) and cytopathic effect (CPE) was evaluated seven days later. Sera samples from uninfected patients (negative controls) and sera samples from confirmed COVID-19 patients (positive controls) were included. Plaque forming units were quantified by direct visualization and the titer of neutralizing antibodies was defined as the highest serum dilution that neutralized 100% of virus infection. Seropositivity rates were calculated as the percentage of the population evaluated that showed end titers ≥1/4 in both techniques.

To assess the cellular immune response, ELISPOT assays were performed using PBMCs from 18 participants, as described previously, using the human IFN-γ/interleukin-4 (IL-4)double-color ELISPOT (Immunospot) (15). Cells were stimulated for 48h in the presence of Mega Pools (MPs) of peptides derived from SARS-CoV-2 Spike WT, Alpha, Beta, Gamma and Delta at 37°C, 5% CO_2_. As positive controls, an independent stimulation performed with 5 mg/mL of Concanavalin A (ConA) (Sigma Life Science #C5275-5MG) and with an MP of peptides derived from cytomegalovirus proteins (MP-CMV) for the stimulation of both CD4^+^ and CD8^+^ T cells. As a vehicle control, DMSO 1% (Merck #317275) was included. Spot Forming Cells (SFCs) were counted on an ImmunoSpot^®^ S6 Micro Analyzer.

### Statistical Analysis

Statistical differences were evaluated by Wilcoxon tests (for comparisons between two groups). Differences were considered significant if the p value was under 0.05. All data were analyzed with GraphPad Prism 9.0.1.

## Results

**Figure 1:**
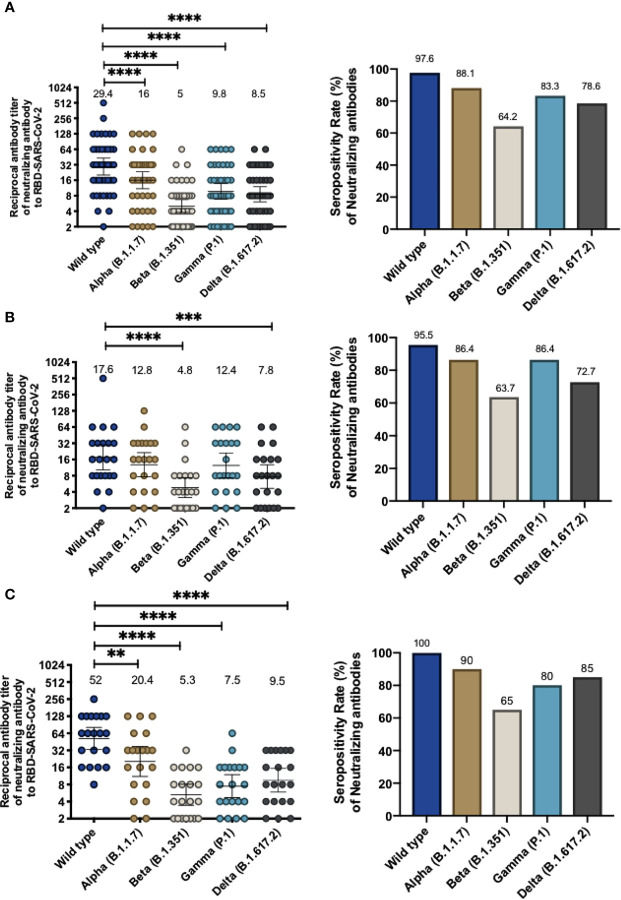
Immunization with CoronaVac induces antibodies able to inhibit the interaction between hACE2 and S1-RBD from SARS-CoV-2 variants after two immunizations in a 0-14 and 0-28 schedule. Antibody titers were evaluated with a surrogate virus neutralization assay (sVNT), which quantifies the interaction between S1-RBD from either WT SARS-CoV-2 or variants of concern (Alpha, Beta, Gamma, and Delta) and hACE2 on ELISA plates. Total neutralizing antibodies titer from volunteers vaccinated with CoronaVac, 28 days after the second dose and the seropositivity rate of neutralizing antibodies are shown for both vaccination schedules **(A)**, 0-14 schedule **(B)** and 0-28 schedule **(C)**. Numbers above the bars show the Geometric Mean Titer (GMT), and the error bars indicate the 95% CI in the graphs showing total antibody titers, and the number above bars show the percentage of seropositivity rate in the respective graphs. A Wilcoxon test analyzed data to compare against the wild-type RBD; **p < 0.005, ***p < 0.001, ****p < 0.0001. The graph represents the results obtained for 22 volunteers for the 0-14 schedule and 20 volunteers for the 0-28 schedule.

To assess whether volunteers from the Phase 3 clinical trial being held in Chile exhibited antibodies able to inhibit the RBD of SARS-CoV-2 circulating variants of concern, we performed an in-house sVNT designed to evaluate the inhibition of the interaction between hACE2 and RBD, which has been previously shown to correlate with neutralizing antibodies (15, 18). Samples from volunteers immunized with two doses of CoronaVac in a 0-14 or 0-28 immunization schedule were tested. Levels of antibodies able to inhibit the interaction between hACE2 and RBD from circulating SARS-CoV-2 variants of concern combining both 0-14 and 0-28 immunization schedules are shown in **Figure 1A**. We report a 1.8-fold reduction of antibody titers that inhibit the variant Alpha, a 5.9-fold reduction of titers against the variant Beta, a 3-fold reduction of titers against the variant Gamma, and a 3.5-fold reduction of titers against the variant Delta, as compared to the WT strain. These reductions were associated with a decrease in GMT values, i.e., 29.5 (95% CI 20.1-43) for the WT strain, 16.0 (95% CI 10.9-23.5) for Alpha, 5.0 (95% CI 3.8-6.7) for Beta, 9.8 (95% CI 6.9-13.9) for Gamma, and 8.5 (95% CI 6.1-11.9) for Delta. Reductions seen for variants Beta, Gamma, and Delta were detected in both age groups. Interestingly, participants aged 18-59 years did not exhibit significant differences in the level of antibodies inhibiting the WT strain and the Alpha variant (**Supplementary Figure 1**). The seropositivity rate of the neutralizing antibodies in the population evaluated was 100% for the WT strain and 88.1%, 64.2%, 88.1% and 78.6% for Alpha, Beta, Gamma, and Delta, respectively.

For the 0-14 immunization schedule, antibodies that inhibit the variants Alpha, Beta, and Gamma were measured 28 days after administration of the second dose. GMTs of antibodies able to inhibit the RBDs (**Figure 1B**) are lower compared to the wild-type strain (17.6, 95% CI 10.2-30.1) and the lowest reported value were against the Beta variant (GMT 4.8, 95% CI 3.1-7.4, a 3.6-fold reduction) and Delta variant (GMT 7.8, 95% CI 4.7-12.9, a 2.3-fold reduction). In contrast, similar GMT values were found for the Alpha and Gamma variants (12.8, 95% CI 7.7-21.5 and 12.4, 95% CI 7.3-21.2, respectively). Similar values were found when samples were analyzed according to their age group, although volunteers aged 18 to 59 years old exhibited a significant decrease in antibodies against the Beta RBD and Delta RBD whereas volunteers over 60 years only exhibit a significant decrease against the Beta RBD (**Supplementary Figures 2A, B**). The seropositivity rate was 95.45% of the evaluated volunteers exhibiting neutralizing antibodies against the WT strain, while the percentages against the Alpha, Beta, Gamma and Delta variants were 86.36%, 63.64%, 86.36%, and 72.72%, respectively.

For volunteers of the 0-28 immunization schedule, increased GMT values in antibodies able to block the RBDs were found against the WT strain (52.0, 95% CI 33.2-81-3) compared to the GMTs for the WT strain observed in the 0-14 schedule, as observed in Fig 1C. These GMT values decreased when evaluating the circulating variants of concern (Alpha, 2.5-fold reduction, GMT 20.4, 95% CI 11.1-37.4; Beta, 9.8-fold reduction, GMT 5.3 95% CI 3.4-8; Gamma, 6.9-fold reduction, GMT 7.5, 95% CI 4.7-11.9; and Delta, 5.5-fold reduction, GMT 9.5 95% CI 5.9-15.4) (**Figure 1C**). Decreases in GMT values against the Beta, Gamma and Delta variants were seen for both age groups in this immunization schedule. However, volunteers aged 18-59 years exhibited a similar GMT between the WT strain and the Alpha variant (**Supplementary Figures 2C, D**). Seropositivity rates of antibodies measured for this schedule are showed in **Figure 1C** and are similar to those reported for the 0-14 schedule. The results indicate that 100% of the evaluated volunteers exhibited antibodies able to inhibit the WT strain, while percentages against the Alpha, Beta, Gamma, and Delta variants were 90%, 65%, 80% and 85%, respectively.

**Figure 2:**
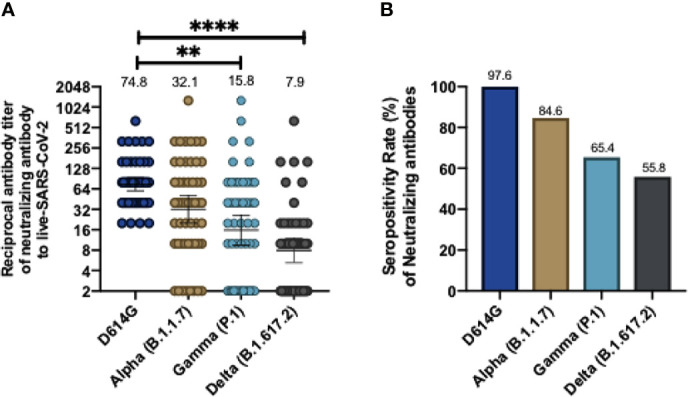
CoronaVac immunization induces neutralizing antibodies against SARS-CoV-2 variants after two vaccine doses using a conventional virus neutralization test. Neutralizing antibody titers were evaluated by incubating the serum with a SARS-CoV-2 Chilean clinical strains and then added into Vero E6 cell for seven days. The neutralizing titer was determinate for the last dilution where no viral cytopathic effect was found in cells against wild type (D614G), and Alpha, Gamma and Delta variants. Consolidate neutralizing antibodies titer of both schedules is shown in **(A)**, and the seropositivity rate of neutralizing antibodies is shown in **(B)**. Numbers above the bars show the Geometric Mean Titer (GMT), and the error bars indicate the 95% CI in **(A)**, and the number above bars in **(B)** showed the seropositivity rate. A Wilcoxon test analyzed data to compare against the wild-type RBD; **p < 0.005, ****p < 0.0001. The graph represents the results obtained for 52 volunteers of both schedules.

In order to further corroborate whether these antibodies were also able to neutralize viral infection in a cell culture, we performed cVNT for lineage B SARS-CoV2 (D614G) and the Alpha, Gamma, and Delta variants. The results obtained showed that, as compared to the D614G strain, there was a 2.33-fold decrease in neutralizing antibodies against the Alpha variant, a 4.73-fold reduction against the Gamma variant and a 9.46-fold reduction against the Delta variant (**Figure 2A**). This result suggests that CoronaVac induce the secretion of antibodies that can neutralize these variants, but at rates lower than those reported for the WT or the D614G strain. The GMT values obtained by cVNT for D614G strain and the Alpha, Gamma, and Delta variants were 74.8 (95% CI 59.8-93.6), 32.1(95% CI 20.1-51.1), 15.8 (95% CI 9.5-26.2) and 7.9 (95% CI 5.2-12), respectively. As also seen for sVNT, volunteers aged 18 to 59 years exhibit a significant decrease in neutralizing antibodies against Gamma, and Delta, whereas volunteers over 60 years old exhibited significantly decreased neutralizing antibodies against Alpha and Delta and a lower but insignificant decrease in neutralizing antibodies against Gamma (**Supplementary Figure 3**). The seropositivity rates of neutralizing antibodies for the Alpha, Gamma and Delta variants were 84.62%, 65.38% and 55.76% respectively, while for the D614G strain was 97.6% (**Figure 2B**). Further details regarding the values reported on **Figures 1** and **2** can be found in **Tables 1** and **2**.

**Table 1:** Seropositivity rates and geometric mean titer of antibodies that inhibit the RBDs of SARS-CoV2 variants, by sVNT.

Schedule	Indicators	Wild type	Alpha (B.1.1.7)	Beta (B.1.351)	Gamma (P.1)	Delta (B.1.617.2)	Seropositivity rate over 2 variants
0-14	Seropositivity n/N (%) GMT (95% CI)	21/22 95.5 17.6 10.3-30.2	19/22 86.4 12.8 7.7-21.5	14/22 63.6 12.4 7.3-21.2	19/22 86.4 4.8 3.2-7.4	16/22 72.72 7.8 4.7-12-9	19/22 86.4 N/D (-)
0-28	Seropositivity n/N (%) GMT (95% CI)	20/20 100 52.0 33.1-81.4	18/20 90.0 20.4 11.1-37.4	13/20 65.0 7.5 4.7-11.2	16/20 80.0 5.3 3.4-8.1	17/20 85.0 9.5 5.9-15.4	18/20 90.0 N/D (-)
Total	Seropositivity n/N (%) GMT (95% CI)	41/42 97.6 29.5 20.2-43.1	37/42 88.1 16.0 10.9-23.5	27/42 64.3 9.8 6.9-13.9	35/42 83.3 5.0 3.8-6.7	33/42 78.57 8.5 6.1-11.9	37/42 88.1 N/D (-)

RBD, Receptor-binding domain; S, Spike; GMT, Geometric mean titer; N/D, Not determined.

**Table 2:** Seropositivity rates and geometric mean titer of neutralizing antibodies against SARS-CoV2 variants by cVNT.

Schedule	Indicators	D614G	Alpha (B.1.1.7)	Gamma (P.1)	Delta (B.1.617.2)	Seropositivity rate over 2 variants
0-14	Seropositivity n/N (%) GMT (95% CI)	34/34 100 57.7 45.1-74.0	27/34 79.4 26.5 14.9-47.1	27/34 79.4 27.0 14.8-49.4	20/34 58.8 7.7 4.7-12-6	29/34 85.2 N/D (-)
0-28	Seropositivity n/N (%) GMT (95% CI)	18/18 100 122.2 83.9-178.1	17/18 94.4 46.1 19.8-107.2	7/18 38.9 5.7 2.6-12.4	9/18 50.0 8.3 3.5-19.7	12/18 66.6 N/D (-)
Total	Seropositivity n/N (%) GMT (95% CI)	52/52 100 74.8 59.8-93.6	44/52 84.6 32.1 20.1-51.1	34/52 65.4 15.8 9.5-26.2	29/52 55.8 7.9 5.2-12	41/52 78.8 N/D (-)

GMT, Geometric mean titer; N/D, Not determined.

**Figure 3:**
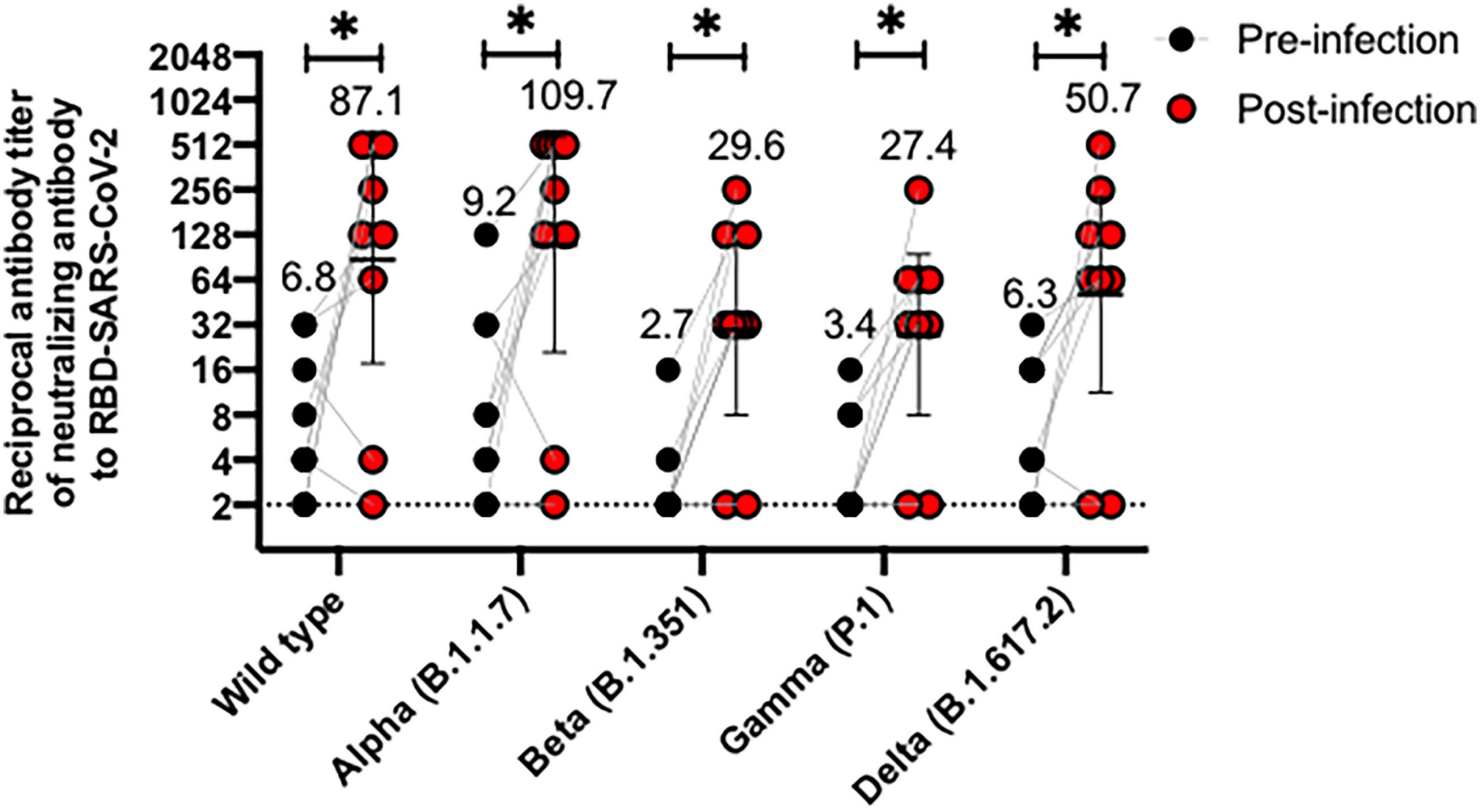
CoronaVac immunization induces antibodies able to inhibit the interaction between hACE2 and S1-RBD from SARS-CoV-2 variants in vaccine breakthrough cases after two vaccine doses. Antibody titers were evaluated with a surrogate virus neutralization assay (sVNT), which quantifies the interaction between S1-RBD from either Wild type SARS-CoV-2 or variants of concern (Alpha, Beta, Gamma, and Delta) and hACE2 on ELISA plates. Comparative data from vaccine breakthrough cases from both schedules are represented for each variant in two different point times, pre-infection (black circle) and post-infection (red circles). A Wilcoxon test analyzed data to compare against the wild-type RBD; *p < 0.05. The graph represents the results obtained for nine volunteers considering both schedules.

We also evaluated whether nine volunteers infected with SARS-CoV-2 after their respective vaccination schedules were completed (breakthrough cases) produced antibodies inhibiting the RBDs of the different variants evaluated. **Figure 3** compares antibodies levels 28 days after the second dose of CoronaVac (pre-infection) and 28 days after the infection were detected (post-infection). Most of the volunteers exhibited a 10-fold increase in the GMT of antibodies able to inhibit the RBDs of the four variants evaluated (Alpha, Beta, Gamma and Delta), as compared to GMT observed for samples previous infection. Therefore, natural infection with SARS-CoV-2 increases the secretion of antibodies that can block the interaction of RBDs from the Beta, Gamma, and Delta variants with the hACE2 receptor. However, further analyses are still required, as no characterization of the variants infecting these volunteers was performed.

**Figure 4:**
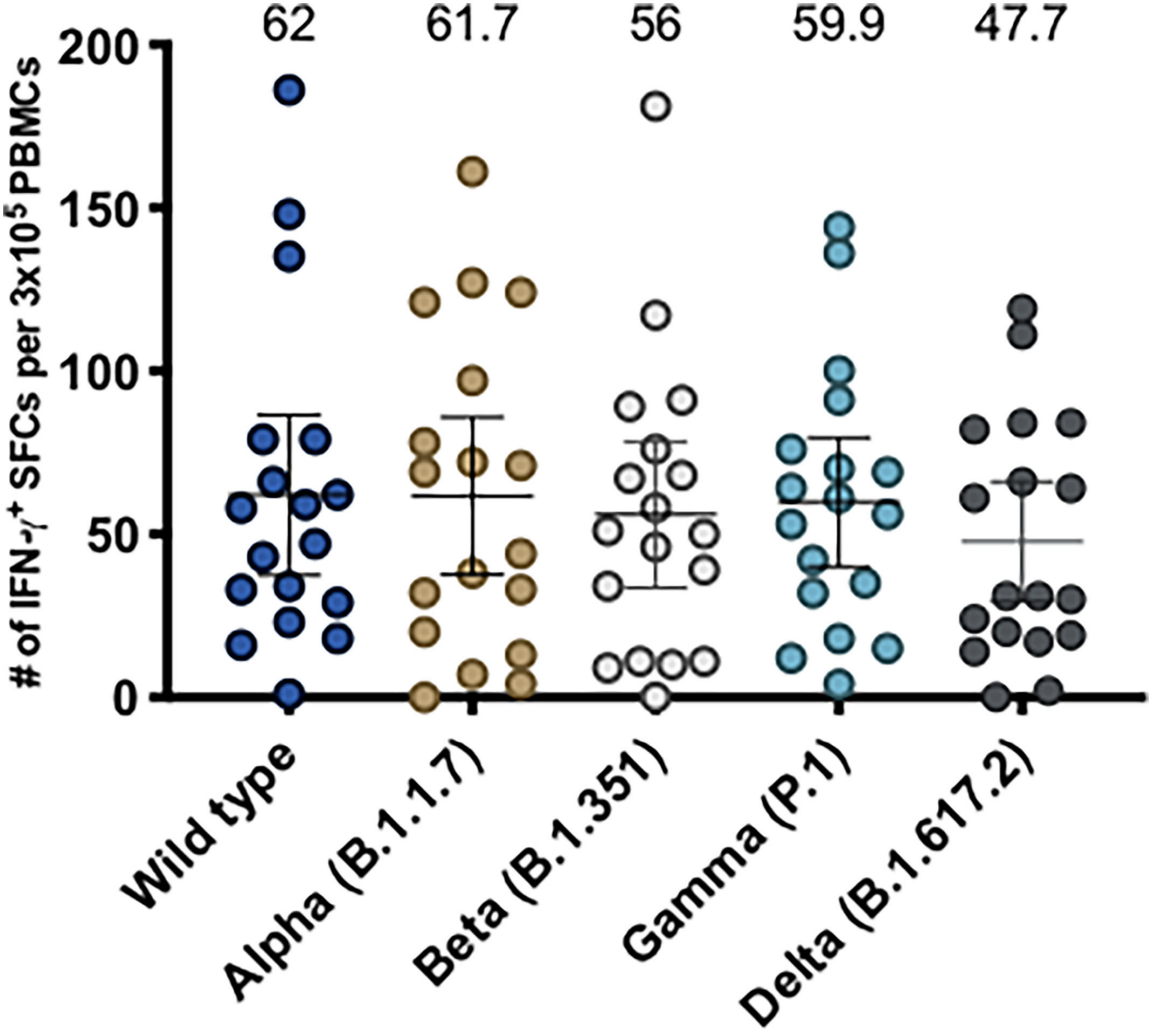
Evaluation of cellular immune response through ELISPOT upon stimulation with Mega Pools of Spike peptides derived from SARS-CoV-2 WT and variants of concern in volunteers immunized with CoronaVac. Numbers of IFN-γ-secreting cells, determined through ELISPOT as spot forming cells (SFCs) were determined. PBMCs were stimulated with MP-S WT, MP-S Alpha, MP-S Beta, MP-S Gamma and MP-S Delta for 48 h for samples obtained 2 weeks after the second dose of volunteers of the 0-14 schedule (n = 11) and 0-28 schedule (n = 7). A total of 18 volunteers were evaluated. Data shown represents mean ± 95% CI and the mean is indicated above each bar. Statistical differences were evaluated by a one-way ANOVA followed by Dunnett’s test for multiple comparisons against the MP-S WT.

Moreover, we have recently shown that CoronaVac is able to stimulate CD4^+^ T cell responses against MPs of both Spike and Non-Spike peptides, displaying higher secretion of IFN-γ and expression of activation markers following vaccination in a 0-14 schedule, which peaks 14 days after the second dose (15). In order to evaluate anti-Spike CD4^+^ T cell responses, we stimulated PBMCs of participants from both 0-14 and 0-28 schedules with Spike MPs from the WT strain and variants of concern and evaluated IFN-γ expression by ELISPOT (**Figure 4**). As previously reported, the subjects evaluated exhibited robust IFN-γ production following stimulation and we did not observe significant differences between PBMCs stimulated with any of the Spike MPs, suggesting that CoronaVac induces protective cellular responses against all SARS-CoV-2 variants of concern. In addition, we observed low numbers of IL-4-secreting T cells in response to all of the MPs (**Supplementary Figure 4**), which is consistent with our previous data using the MP-S WT.

## Discussion

The current spread of multiple SARS-CoV-2 variants worldwide challenges the strategies of vaccination and represent a threat for potential new waves of infection. The inactivated SARS-CoV-2 vaccine CoronaVac has been proven to induce total IgG and neutralizing antibodies against the Spike protein in subjects vaccinated with either a 0-14 or 0-28 vaccination schedule, although those levels are lower as compared to other vaccines such as BNT16b2 and Moderna mRNA-1273 (15, 19, 20). Here we report that CoronaVac induces the secretion of neutralizing antibodies that recognize most of the variants of concern currently circulating in the population, as determined by sVNT and cVNT (**Figures 1**–**3**). Although the intrinsic characteristics for each of the techniques used in this report to evaluate circulating neutralizing antibodies in immunized volunteers were different, the results obtained were mostly equivalent for the WT strain, as described in our previous studies (15, 21). We found similar fold reductions in blocking and neutralizing antibodies against the variants Alpha and Gamma using both techniques, but a higher fold reduction against the Delta variant (3.5-fold reduction in the sVNT and 9.46-fold reduction in the cVNT) was observed. Moreover, when evaluating through cVNT, lower seropositivity rates were observed against the Gamma and Delta variants (65.4% and 55.8%, respectively) as compared to the results obtained by sVNT (83.3% and 78.57%, respectively), but we report a similar percentage of seropositivity for participants with circulating neutralizing antibodies against at least two of the variants with both techniques (88.1% by sVNT and 78.8 by cVNT) (**Tables 1** and **2**). These results are in line with previous reports that have shown a high correlation between these two techniques (15, 18). A recent study that used the sVNT and cVNT to evaluate neutralizing antibodies against SARS-CoV-2 variants of concern in heterologous and homologous ChAdOx1 nCoV-19/BNT162b2 vaccination has shown high correlation between both assays (22).

Our results are in line with the effectiveness of CoronaVac observed in a study of elderly subjects vaccinated in Brazil, where the Gamma variant is the most prevalent SARS-CoV-2 strain and an effectiveness of 42% was reported (23). Furthermore, our data is consistent with a recent study in volunteers vaccinated with two doses of CoronaVac in China, which exhibit a 4.3-fold reduction of VNT in live neutralization assays against the Gamma variant compared to the WT strain and another study with individuals vaccinated with two doses of CoronaVac in Brazil, which reported reduced VNT against the isolates P.1/28 and P.1/30 as compared to the WT strain (a 3.1 and 2.6 fold reduction, respectively) (24, 25). Similarly, here we report a 4.73 fold reduction compared to the D614G strain using cVNT (**Figure 2**). In addition, other studies carried out in Chile using cVNT and pseudotyped viruses have reported a 7.51 and 2.33-fold reduction, respectively, in Gamma variant neutralization as compared to the WT strain in subjects vaccinated with CoronaVac (26, 27). The reduced neutralizing capacities reported against the Gamma variant have been related to the E484K mutation, which promotes the evasion of neutralizing antibodies (28). Importantly, the Gamma variant became one of the dominant SARS-CoV-2 strains in Chile during 2021 in parallel to the vaccination of Chilean population with CoronaVac (26). However, only 45 out of 2,263 participants of the phase 3 clinical trial carried out in Chile developed breakthrough cases following vaccination and among these individuals 96% developed mild disease, which suggests that CoronaVac is protective against SARS-CoV-2 and potentially against SARS-CoV-2 variants (21).

We also reported neutralizing responses against the Beta variant in subjects vaccinated with two doses of CoronaVac. A reduced inhibition of the interaction between hACE2 and RBD compared to the WT strain and a seropositivity of 64.2% was reported using the sVNT, the lowest across all variants of concern analyzed (**Figure 1** and **Table 1**). These results are consistent with recent reports in cohorts from Thailand and China vaccinated with CoronaVac, in which reduced neutralization was reported using live virus neutralization (fold reductions of 22.1 and 5.7 compared to the WT strain, respectively) (24, 29) and also with the reduction in neutralizing responses observed in subjects vaccinated with the mRNA vaccine BNT162b2 for the Beta variant (4, 30). In line with the reports for the Gamma variant, the E484K mutation found in the Beta variant has been identified as the main mutation responsible for this effect as antibodies bind to RBD with less affinity.

Of note, we used the D614G variant in the cVNT, which exhibits a mutation outside of the RBD and we were able to observe effective neutralization against viral infection in all the subjects evaluated from both vaccination schedules and both age groups (**Figure 2**). These results support that CoronaVac is protective against the D614G variant, which is one of the most prevalent strains worldwide.

Our work also reported protection against the variant Delta. The Delta variant (first identified in India) exhibit the RBD mutations T478K, L452R and P681R and is currently a cause of concern due to its high transmissibility and may even surpass other variants in this regard (11). The Delta variant has been recently detected in Chile and it is becoming one of the dominant SARS-CoV-2 strains. Here we show using a RBD containing the mutations T478K and L452R present in the Delta variant that volunteers vaccinated with CoronaVac exhibit reduced blocking antibodies compared to the WT RBD but we report a seropositivity of 78.57% and 55.76% by sVNT and cVNT (**Tables 1** and **2**), respectively, which suggests that the vaccine confers protection against this variant. Our data is in line with the previously mentioned works from Thailand and China in volunteers vaccinated with 2 doses of CoronaVac, in which neutralization was evaluated by cVNT and reported fold reductions of 31.7 and 3.7 fold reduction, respectively, as compared to the WT strain, whereas we report a 9.46-fold reduction (24, 29). Similarly, mRNA vaccines induce neutralizing antibodies against the Delta variant but to a reduced extent compared to the WT strain (31, 32). Pseudoviruses carrying the L452R mutation display higher infectivity in cell culture and when incubated with sera from subjects vaccinated with Moderna mRNA-1273 or BNT16b2, as compared to the WT strain (13).

Our study also shows how subjects vaccinated with CoronaVac increase their blocking antibody GMTs following natural infection against the wild type strain and to a similar extent to the Alpha variant, but this increased GMT was lower for the variants Beta, Gamma and Delta (**Figure 3**). These findings are consistent with studies comparing different vaccine platforms against natural infection, which indicate that inactivated vaccines induce lower levels of neutralizing antibodies compared to natural infection with SARS-CoV-2, in contrast to mRNA vaccines, which exhibit comparable levels of neutralization, using live virus neutralization (20). In line with this, cohorts from Thailand and Brazil vaccinated with CoronaVac exhibits lower neutralizing antibody titers against either the WT strain or variants of concern, compared to naturally infected individuals (25, 29). We have previously reported levels of neutralization in unvaccinated and naturally infected hospitalized individuals, which exhibit a robust neutralizing antibody response against wild-type SARS-CoV-2 (33). Although we did not perform cVNT for either breakthrough cases or naturally infected individuals against variants of concern, our results obtained by sVNT are in line with data from non-variant infected subjects, who also exhibit a similar reduction in neutralization against the variants Beta, Gamma and Delta (20).

Moreover, here we show that CoronaVac is able to stimulate T cell responses against Spike MPs from either WT strain or variants of concern and we did not see any significant differences (**Figure 4**). This is the first report to date to characterize T cell responses against SARS-CoV-2 Spike MPs in volunteers vaccinated with CoronaVac. Concordantly, MPs from variants of concern have been previously used to show that volunteers vaccinated with two doses of either Moderna mRNA-1273 or BNT16b2 exhibit IFN-γ-secreting T cells in response to these MPs and no significant differences were found (34). These results have been attributed to the high conservation of T cell epitopes in variants of concern, suggesting that vaccines can induce effective cellular responses against them. In addition, it is important to highlight that although the majority of the T cell responses are conserved and the variants do not mutate enough to disrupt the overall T cell repertoire, mutations are observed in other SARS-CoV-2 proteins and across variants (34). Therefore, it is likely that the induction of cellular responses against other SARS-CoV-2 proteins by CoronaVac may confer an advantage compared to other vaccines, considering that the inclusion of multiple antigens might increase the likelihood that more epitopes are conserved than having only one protein in the vaccine.

Importantly, a limitation of our study is that we were not able to characterize other non-neutralizing antibody functions that could be important in either vaccinated or convalescent subjects against variants of concern. Furthermore, *in vitro* evaluation of neutralizing antibodies does not necessarily correlate with protection against SARS-CoV-2 in vaccinated individuals. However, recent evidence supports that levels of neutralizing antibodies are predictive of protection against symptomatic SARS-CoV-2 infection (35). In addition, although cellular responses do not necessarily prevent infection, induction of cellular responses against variants of concern in individuals vaccinated with CoronaVac suggests that vaccinated individuals are protected from severe disease, which is supported from the results of the clinical trial performed in Chile with this vaccine (16, 21).

## Data Availability Statement

The raw data supporting the conclusions of this article will be made available by the authors, without undue reservation.

## Ethics Statement

This trial was approved by each Institutional Ethical Committee and the Chilean Public Health Institute (#24204/20) and conducted according to the current Tripartite Guidelines for Good Clinical Practices, the Declaration of Helsinki. The patients/participants provided their written informed consent to participate in this study.

## Author Contributions

Conceptualization and visualization, AK, ER, SB, KA, PG, and JG-A. Methodology, RF, JM, JF, GZ, WM, AG, AS, and DW. Investigation, FM-G, JS, JF, NB, LG, BS, LD, NG, GAP, RB-R, GH-E, CI, DM-T, MR, DR-P, OV, MU, and YV. Funding acquisition, AK. Project administration, AK, KA, SB, PG, and JG-A. Supervision, AK, KA, SB, and PG. Writing – original draft, FM-G and JS. Writing – review and editing, AK, KA, SB, ER, PG, AG, AS, and DW. All authors contributed to the article and approved the submitted version.

## Funding

This work was supported by The Ministry of Health, Government of Chile supported the funding of the CoronaVac03CL Study, The Confederation of Production and Commerce (CPC), Chile, supported the funding of the CoronaVac03CL Study, the NIH NIAID under Contract No. 75N93021C00016 supports AS and Contract No. 75N9301900065 supports AS, DW. The Millennium Institute on Immunology and Immunotherapy, ANID - Millennium Science Initiative Program ICN09_016 (former P09/016-F) supports SB, KA, PG, and AK. The Innovation Fund for Competitiveness FIC-R 2017 (BIP Code: 30488811-0) supports SB, PG, and AK. SINOVAC contributed to this study with the investigational vaccine and experimental reagents.

## Conflict of Interest

Authors GZ and WM are employed by company SINOVAC Biotech. AS is a consultant for Gritstone Bio, Flow Pharma, Arcturus Therapeutics, ImmunoScape, CellCarta, Avalia, Moderna, Fortress and Repertoire.

The remaining authors declare that the research was conducted in the absence of any commercial or financial relationships that could be construed as a potential conflict of interest.

La Jolla Institute for Immunology (LJI) has filed for patent protection for various aspects of T cell epitope and vaccine design work.

The authors declare this study received the investigational product (placebo and vaccines) from the company SINOVAC Biotech. SINOVAC employees contributed to the conceptualization of the study (clinical protocol and eCRF design) but did not participate in either the analysis or interpretation of the data shown in this manuscript.

## Publisher’s Note

All claims expressed in this article are solely those of the authors and do not necessarily represent those of their affiliated organizations, or those of the publisher, the editors and the reviewers. Any product that may be evaluated in this article, or claim that may be made by its manufacturer, is not guaranteed or endorsed by the publisher.

## Supplementary Material

Supplementary Figure S1 Immunization with CoronaVac induces antibodies able to inhibit the interaction between hACE2 and S1-RBD from SARS-CoV-2 variants in participants aged 18-59 and ≥60 after two immunizations. Antibody titers were evaluated with a surrogate virus neutralization assay, which quantifies the interaction between S1-RBD from either Wild type SARS-CoV-2 or variants of concern (Alpha, Beta, Gamma and Delta) and hACE2 on ELISA plates. Results were obtained from participants vaccinated with CoronaVac, 28 days after the second dose in volunteers between 18-59 **(A)** and ≥ 60 **(B)** consolidating the data from both 0-14 and 0-28 schedules. Numbers above the bars show the Geometric Mean Titer (GMT), and the error bars indicate the 95% CI. A Wilcoxon test analyzed data to compare against the wild-type RBD; ****p < 0.0001. The graph represents the results obtained for 22 participants in the 18-59 years old group and 20 participants in the ≥60 years old group.

Supplementary Figure S2 CoronaVac vaccination induces antibodies able to inhibit the interaction between hACE2 and S1-RBD from SARS-CoV-2 variants in participants aged 18-59 and ≥60 after two immunizations in both 0-14 and 0-28 schedules. Antibody titers were evaluated with a surrogate virus neutralization assay, which quantifies the interaction between S1-RBD from either Wild type SARS-CoV-2 or variants of concern (Alpha, Beta, Gamma and Delta) and hACE2 on ELISA plates. Results were obtained from participants vaccinated with CoronaVac 28 days after the second dose. For 0-14 schedule, volunteers between 18-59 and ≥ 60 are shown in **(A, B)**, respectively, and for 0-28, schedule volunteers between 18-59 and ≥ 60 are shown in **(C, D)**, respectively. The bars above show the Geometric Mean Titer (GMT), and the error bars indicate the 95% CI. A Wilcoxon test analyzed data to compare against the wild-type RBD; **p < 0.05, ***p < 0.005, ****p < 0.0001. The graph represents the results obtained for 12 participants in the 18-59 years old group and 10 participants in the ≥60 years old group in the 0-14 schedule and for 10 participants in the 18-59 years old group and 10 participants in the ≥60 years old group in the 0- 28 schedule.

Supplementary Figure S3 CoronaVac immunization induces neutralizing antibodies against SARS-CoV-2 variants after two vaccine doses using a live virus test in volunteers aged 18-59 and over 60 years old. Antibody titers were evaluated by incubating the serum with a SARS-CoV-2 Chilean clinical strain and then added into Vero E6 cell for seven days. The neutralizing titer was determinate for the last dilution where no viralcytopathic effect was found in cells against wild type (D614G) and Alpha, Gamma and Delta variants. Consolidate neutralizing antibodies titer of volunteers from 0-14 and 0-28 schedules aged 18-59 years old are shown in **(A)**, while volunteer under 60 years old from 0-14 and 0-28 schedules are shown in **(B)**. The bars above show the Geometric Mean Titer (GMT), and the error bars indicate the 95% CI. A Wilcoxon test analysed data to compare against the wild-type RBD; *p < 0.05. The graph represents the results obtained for 42 volunteers of both schedules.

Supplementary Figure S4 Evaluation of cellular immune response through ELISPOT upon stimulation with Mega Pools of Spike peptides derived from SARS-CoV-2 WT and variants of concern in volunteers immunized with CoronaVac. Numbers of IL-4-secreting cells, determined through ELISPOT as spot forming cells (SFCs) were determined. PBMCs were stimulated with MP-S WT, MP-S Alpha, MP-S Beta, MP-S Gamma and MP-Delta for 48 h for samples obtained 2 weeks after the second dose of volunteers of the 0-14 schedule (n = 11) and 0-28 schedule (n = 7). A total of 18 volunteers were evaluated. Data shown represents mean 95% CI and the mean is indicated above each bar. Statistical differences were evaluated by a one-way ANOVA followed by Dunnett’s test for multiple comparisons against the MP-S WT.
