# Supplementary figures and images for: Recognition of Variants of Concern by Antibodies and T Cells Induced by a SARS-CoV-2 Inactivated Vaccine
Source: Front Immunol. 2021 Nov 9;12:747830. doi: 10.3389/fimmu.2021.747830 (PMC8630786; doi:10.3389/fimmu.2021.747830)

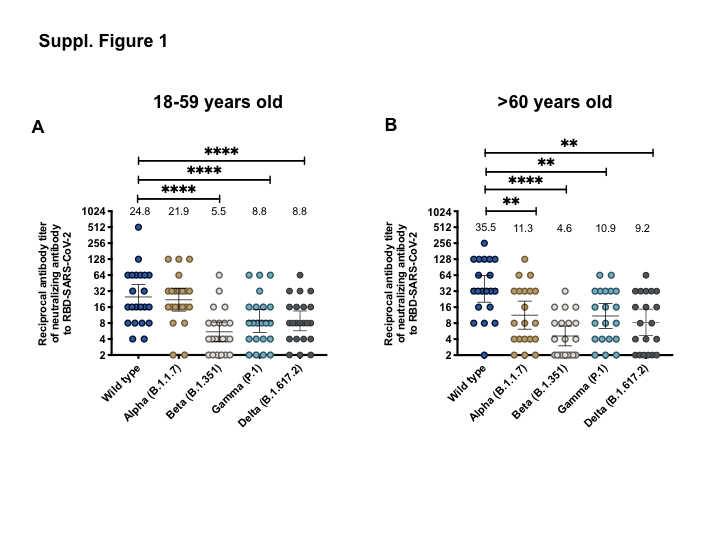

Supplement: Supplementary Figure S1 — Immunization with CoronaVac induces antibodies able to inhibit the interaction between hACE2 and S1-RBD from SARS-CoV-2 variants in participants aged 18-59 and ≥60 after two immunizations. Antibody titers were evaluated with a surrogate virus neutralization assay, which quantifies the interaction between S1-RBD from either Wild type SARS-CoV-2 or variants of concern (Alpha, Beta, Gamma and Delta) and hACE2 on ELISA plates. Results were obtained from participants vaccinated with CoronaVac, 28 days after the second dose in volunteers between 18-59 (A) and ≥ 60 (B) consolidating the data from both 0-14 and 0-28 schedules. Numbers above the bars show the Geometric Mean Titer (GMT), and the error bars indicate the 95% CI. A Wilcoxon test analyzed data to compare against the wild-type RBD; ****p < 0.0001. The graph represents the results obtained for 22 participants in the 18-59 years old group and 20 participants in the ≥60 years old group. [file Image_1.tiff]

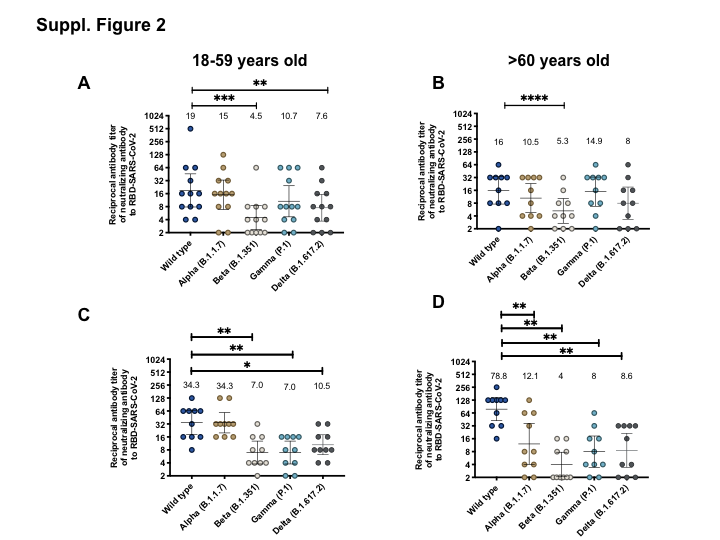

Supplement: Supplementary Figure S2 — CoronaVac vaccination induces antibodies able to inhibit the interaction between hACE2 and S1-RBD from SARS-CoV-2 variants in participants aged 18-59 and ≥60 after two immunizations in both 0-14 and 0-28 schedules. Antibody titers were evaluated with a surrogate virus neutralization assay, which quantifies the interaction between S1-RBD from either Wild type SARS-CoV-2 or variants of concern (Alpha, Beta, Gamma and Delta) and hACE2 on ELISA plates. Results were obtained from participants vaccinated with CoronaVac 28 days after the second dose. For 0-14 schedule, volunteers between 18-59 and ≥ 60 are shown in (A, B), respectively, and for 0-28, schedule volunteers between 18-59 and ≥ 60 are shown in (C, D), respectively. The bars above show the Geometric Mean Titer (GMT), and the error bars indicate the 95% CI. A Wilcoxon test analyzed data to compare against the wild-type RBD; **p < 0.05, ***p < 0.005, ****p < 0.0001. The graph represents the results obtained for 12 participants in the 18-59 years old group and 10 participants in the ≥60 years old group in the 0-14 schedule and for 10 participants in the 18-59 years old group and 10 participants in the ≥60 years old group in the 0- 28 schedule. [file Image_2.tiff]

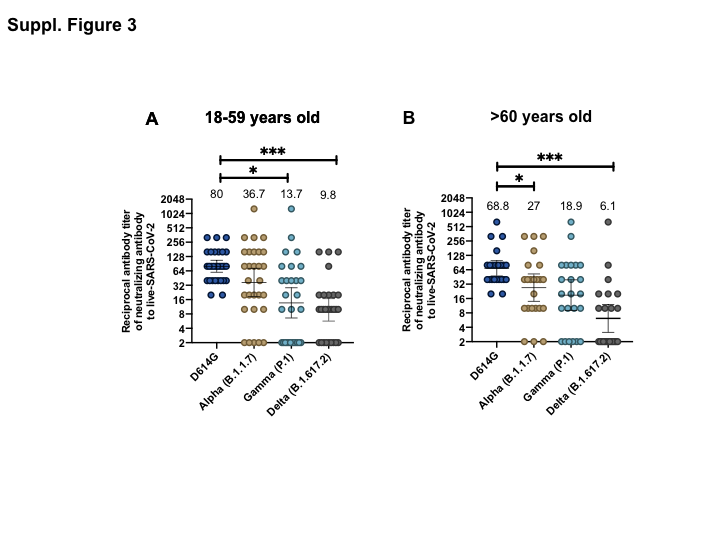

Supplement: Supplementary Figure S3 — CoronaVac immunization induces neutralizing antibodies against SARS-CoV-2 variants after two vaccine doses using a live virus test in volunteers aged 18-59 and over 60 years old. Antibody titers were evaluated by incubating the serum with a SARS-CoV-2 Chilean clinical strain and then added into Vero E6 cell for seven days. The neutralizing titer was determinate for the last dilution where no viralcytopathic effect was found in cells against wild type (D614G) and Alpha, Gamma and Delta variants. Consolidate neutralizing antibodies titer of volunteers from 0-14 and 0-28 schedules aged 18-59 years old are shown in (A), while volunteer under 60 years old from 0-14 and 0-28 schedules are shown in (B). The bars above show the Geometric Mean Titer (GMT), and the error bars indicate the 95% CI. A Wilcoxon test analysed data to compare against the wild-type RBD; *p < 0.05. The graph represents the results obtained for 42 volunteers of both schedules. [file Image_3.tiff]

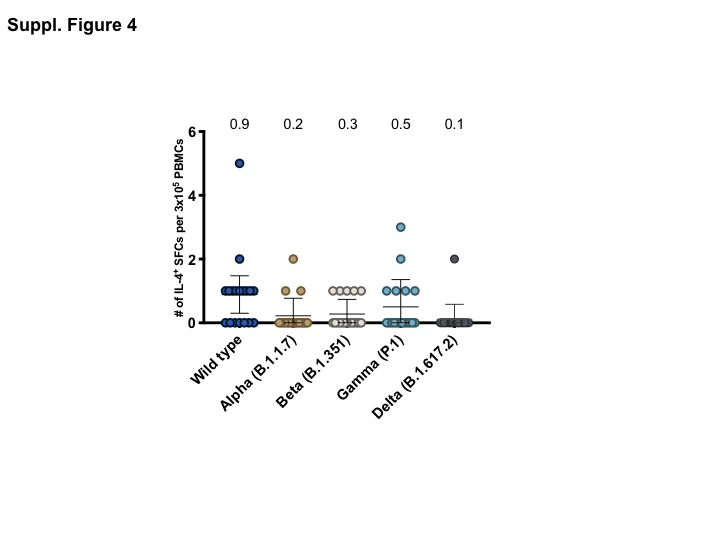

Supplement: Supplementary Figure S4 — Evaluation of cellular immune response through ELISPOT upon stimulation with Mega Pools of Spike peptides derived from SARS-CoV-2 WT and variants of concern in volunteers immunized with CoronaVac. Numbers of IL-4-secreting cells, determined through ELISPOT as spot forming cells (SFCs) were determined. PBMCs were stimulated with MP-S WT, MP-S Alpha, MP-S Beta, MP-S Gamma and MP-Delta for 48 h for samples obtained 2 weeks after the second dose of volunteers of the 0-14 schedule (n = 11) and 0-28 schedule (n = 7). A total of 18 volunteers were evaluated. Data shown represents mean 95% CI and the mean is indicated above each bar. Statistical differences were evaluated by a one-way ANOVA followed by Dunnett’s test for multiple comparisons against the MP-S WT. [file Image_4.tiff]
